# Navigating patients with atopic dermatitis or chronic spontaneous urticaria during the COVID-19 pandemic

**DOI:** 10.3389/falgy.2022.809646

**Published:** 2022-10-04

**Authors:** Isabelle Haddad, Kathia Kozman, Abdul-Ghani Kibbi

**Affiliations:** Department of Dermatology, American University of Beirut Medical Center, Beirut, Lebanon

**Keywords:** skin allergies, atopic dermatitis, chronic urticaria, COVID-19, SARS-CoV-2, pandemic

## Abstract

A rapid spread of different strains of the severe acute respiratory syndrome coronavirus 2 (SARS-CoV-2) has led to an unprecedented pandemic. Since the onset of the coronavirus disease 2019 (COVID-19) pandemic, the medical body has encountered major obstacles concerning disease management at different levels. Even though patients infected with this virus mainly present with respiratory symptoms, it has been associated with a plethora of well-documented cutaneous manifestations in the literature. However, little investigations have been conducted concerning COVID-19 and its impact on skin disorders mediated by type 2 inflammation leaving multiple dermatologists and other specialists perplexed by the lack of clinical guidelines or pathways. This review focuses on the effects of this pandemic in patients with skin disorders mediated by type 2 inflammation, specifically atopic dermatitis and chronic spontaneous urticaria. In addition, it will provide clinicians a guide on treatment and vaccination considerations for this stated set of patients.

## Introduction

Given the rapid spread of the SARS-CoV-2 worldwide, multiple nations adopted a lockdown policy limiting all types of activities including healthcare services. Therefore, this incurred alternative approaches to maintain continuity of care including increasing use of teledermatology and home-based treatments (1). This was simultaneously accompanied by exercising sanitary practices, particularly hand hygiene (2). These measures affected the care of patients with chronic diseases such as atopic dermatitis and chronic urticaria among others and has led to an acute need of developing practical guidelines for the management of these conditions (1, 2). These have been mostly challenging in patients treated with systemic immunotherapies (1–3).

The aim of this review is to highlight the impact of COVID-19 on patients with atopic dermatitis and chronic urticaria as well as provide clinicians with a guide on navigating these patients regarding treatment and vaccinations throughout the pandemic.

## Atopic dermatitis

### Impact of COVID-19

Atopic dermatitis (AD) is a complex disease caused by the interplay of genes, immune system and the environment. It typically develops during infancy or childhood and is characterized by intense pruritus and a chronic or chronically relapsing dermatosis. Management of AD may be quite challenging and requires adequate continuity of care, especially in the older age group (4, 5).

Literature and real-world experience have indicated that COVID-19 poses a great risk on patients with comorbidities including patients with atopic dermatitis (3). This is attributed to the fact that atopic dermatitis is often associated with other atopic disorders such as asthma and allergic rhino-conjunctivitis (1). Studies did not demonstrate atopic dermatitis as a key independent risk factor to developing severe SARS-CoV-2 infection (5–7). However, more recent studies established that AD is associated with an increased odds of COVID-19 infection even after controlling for common comorbidities (8, 9). A systematic review evaluating cutaneous manifestations in patients infected with COVID-19 have shown that 20% of these patients had an underlying dermatologic disease including atopic dermatitis (10). The most common cutaneous presentation in this cohort of patients has been a vesicular rash with no predilection for location (10). Unfortunately, and until date, it is unclear how this virus pathogenetically affects AD patients (3).

The exaggerated immune innate and impaired adaptive immune inflammatory responses causing a cytokine storm have been postulated as a potential mechanism/s by which SARS-CoV-2 causes severe illness (10). Given the fact that AD is an immune-mediated disease, an associated surge in flares of atopic dermatitis has been observed during the pandemic (11). It is imperative then to assume that strict hygiene practices and increased stress levels imposed by the pandemic will likely contribute to this noted increase in AD flares (2, 5, 12). In addition, the pandemic with the need to quarantine has negatively impacted the quality of life of AD patients with reported increased levels of anxiety and depression in this specific subset (7, 12, 13). Physicians should take into account these issues and should incorporate this psychosocial dimension in their management plan.

### Treatment considerations

The lockdown measures put in place during this critical COVID-19 pandemic has markedly decreased clinical activities in dermatology clinics and limited access to care by patients. However, it also gave health care professionals a window of opportunity in the field of teledermatology (web- and phone- counseling) which has significantly surged during the pandemic (14, 15). This platform has been and will likely be an extremely useful mean of reducing patient access to hospital (2, 5). From a patient's perspective, teledermatology has been well-accepted by AD patients who continued to have access to a dermatologist consultation, guaranteeing support and treatment continuation in most cases (1, 5, 16).

To minimize AD flares, the American Academy of Dermatology (AAD) and the European Task Force on Atopic Dermatitis (ETFAD) advocated reinforcing proper skin care regimens. These include the use of fragrance-free soap with warm water for hand washing followed directly by the application of moisturizer. If soap is unavailable, patients with AD should be instructed to use hand sanitizers devoid of sensitizing ingredients to minimize the risk of developing allergic contact dermatitis. Concerning face masks, cotton-woven face cloths are preferred over other potentially irritating face masks. Frequent laundering of these face cloths is recommended though studies on the optimal frequency of washing and the precise make of the fabric are currently lacking and will require additional studies (2, 3).

Treatment of AD may be complex and necessitate the employment of immune-modulating therapies. Such therapies may be of concern if initiated in the setting of SARS-CoV-2 given the dysfunctional and/or exaggerated immune response seen with this infection (10). Additionally, this poses a challenge for AD patients who are maintained on immune-modulating therapies since there is a concern of an increased risk of infection (1, 3). With the multitude of therapeutic modalities currently employed to control moderate to severe AD including systemic corticosteroids, dupilumab, methotrexate, cyclosporine, azathioprine, Janus kinase (JAK) inhibitors and phototherapy among others, recommendations concerning their use during the pandemic have been outlined by international and national societies and are summarized in (Table 1) (17). However, an interdisciplinary approach for severe AD cases is favored (3).

**Table 1:** Summary of current recommendation for nonbiologic and biologic systemic medications in AD treatment.

Treatment	Mechanism of action	Recommendation
Systemic corticosteroids	Non-biologic, conventional systemic broad-spectrum immunosuppressive therapy.	• Without confirmed COVID-19 infection: effect on severity of COVID-19 is still unknown. Taper to lowest effective dose (1–3, 17). • With confirmed COVID-19 infection: treatment should be discontinued because it can increase the risk of infections (1, 2, 18). • Caution regarding initiation and dupilumab is preferred in such cases (1, 2, 17).
Dupilumab	Biological therapy. It blocks the IL-4/IL-13 receptor chain which inhibits Th2 inflammation. It does not impair the immune host response against viral infections.	• Without confirmed COVID-19 infection: treatment continued and might be preferred in selected severe cases than conventional treatment since it is not considered to increase the risk for viral infections (1–3, 17, 18). • With confirmed COVID-19 infection: treatment should be stopped for a minimum of 2 weeks until recovery and/ or a documented negative swab analysis for SARS-Cov-2 (2, 3, 17, 18). • Patient can be initiated safely on this regimen (1, 2, 17).
Methotrexate	Non-biologic, conventional systemic immunosuppressive therapy. It inhibits the synthesis of nitrogenous bases leading to a halt in the proliferation of T and B lymphocytes.	• Without confirmed COVID-19 infection: effect on severity of COVID-19 is still unknown. Taper to lowest effective dose (1–3, 17). • With confirmed COVID-19 infection: treatment should be discontinued because it can increase the risk of infections (2, 3, 17). • Caution regarding initiation and dupilumab is preferred in such cases (1, 2, 17).
Cyclosporine	Non-biologic, conventional systemic immunosuppressive therapy. It is a calcineurin inhibitor which will inhibit TCR signaling and therefore inhibits the activation of T helper lymphocytes.	• Without confirmed COVID-19 infection: effect on severity of COVID-19 is still unknown. Taper to lowest effective dose (1–3, 17). • With confirmed COVID-19 infection: treatment should be discontinued because it can increase the risk of infections (2, 3, 17). • Caution regarding initiation and dupilumab is preferred in such cases (1, 2, 17).
Azathioprine	Nonbiologic, conventional systemic immunosuppressive therapy. It inhibits T and B lymphocyte proliferation.	• Without confirmed COVID-19 infection: effect on severity of COVID-19 is still unknown with no data on its safety profile. Taper to lowest effective dose (1–3, 17). • With confirmed COVID-19 infection: treatment should be discontinued because it can increase the risk of infections (2, 3, 17). • Caution regarding initiation and dupilumab is preferred in such cases (1, 2, 17).
JAK inhibitors	Biological therapy. It inhibits the ATP-binding site of JAK thus inhibiting the enzyme's activity and suppressing subsequent signal transduction.	• Without confirmed COVID-19 infection: treatment can be continued (2). • With confirmed COVID-19 infection: discontinuation during initial infection. However, a potential treatment role for inhibiting the cytokine release is under investigation (2). • No clear data about safety of initiating therapy
Phototherapy	Non-biological therapy.	Discontinued to limit patient's exposure (1, 3).

If systemic therapy needs to be discontinued, the clinician should focus on maximizing the use of natural sunlight, bleach baths, moisturizers, topical therapies, and wet wraps to maintain disease control (1–3, 17).

An international effort to facilitate and guide clinical decision making during the pandemic has been launched *via* SECURE-AD (Surveillance Epidemiology of Coronavirus Under Research Exclusion—Atopic Dermatitis) (www.covidderm.org). It is a web-based registry for clinicians to report COVID-19 outcomes in atopic dermatitis patients (20). This large dataset will serve as a reference for health care professionals and will provide counseling and information about treatment/s during the COVID-19 pandemic. Also, it will aid in filling our present knowledge gaps in regards to the management of AD.

### Vaccination considerations

To achieve herd immunity against the virus, safe and effective vaccines have been developed. The current available vaccines are either mRNA or virus vectored vaccines. There is no evidence to suggest that any will pose a risk of disease flares in patients with AD receiving any of the SARS-CoV-2 vaccine given the fact that response to such vaccines is primarily a Th1 response (21). Even though the chronic phase of AD is a Th2-mediated process, there is currently no data on Th2 response following vaccination (22). Hence, there is no contraindication to vaccination in the setting of AD (6, 21).

In regards to patients on systemic immunosuppressants and JAK-inhibitors, these medications may attenuate or blunt the expected immune response (6, 23). Therefore, it has been recommended to either pause therapy or reduce dosing depending on the systemic therapeutic modality used during vaccination to achieve an optimal immune response (Table 2) (6, 23). Although dupilumab is a biologic therapy for AD, no attenuation is expected (6, 21, 23). Noteworthy is the ETFAD recommendation to schedule the vaccine in the week interval between dupilumab injections (6). Moreover, it is prudent to have the injection of dupilumab at least 48–72 h apart from that of the vaccine in order to distinguish any potential adverse reaction occurring after dupilumab from those occurring after the vaccine. As for the pediatric population, the Food and Drug Administration (FDA) and EMA (European Medicines Agency) have approved the administration of COVID-19 vaccines (Pfizer-BioNTech, Moderna, Novanax) from the age of 6 months to 17 years. At present, there are no studies assessing the impact of the vaccine/s on AD in terms of triggering it or affecting its course in patients who have it.

**Table 2:** Summary of current recommendation for SARS-CoV-2 vaccination in AD patients maintained on systemic therapy.

Treatment	Recommendation
Systemic corticosteroids	Either taper to lowest effective dose or pause treatment for 2 weeks since vaccination day (6, 21).
Methotrexate	Either taper to lowest effective dose or pause treatment for 2 weeks since vaccination day (6, 21).
Cyclosporine	Either taper to lowest effective dose or pause treatment 1 week since vaccination day (6, 21).
Azathioprine	Either taper to lowest effective dose or pause treatment for 2 weeks since vaccination day (6, 21).
JAK inhibitors	Conflicting data: 1 week (6) or no need to pause treatment (23).

## Chronic spontaneous urticaria

### Impact of COVID-19

Chronic spontaneous urticaria (CSU) is an autoimmune/autoallergic condition with a fluctuating course and may be difficult to control. It is characterized by the appearance of recurrent evanescent wheals lasting for more than 6 weeks and variable degree of pruritus. During the COVID-19 pandemic, several case reports, and series have been published on acute urticaria as a cutaneous manifestation of the SARS-CoV-2 infection (24). A third of patients with CSU experiencing exacerbation of their condition have been reported when infected with the virus (24–26). This exacerbation may most likely be seen in patients with moderate-to-severe forms of the infection (25). In terms of pathogenesis, the SARS-CoV-2 virus is postulated to increase pro-inflammatory cytokines causing direct mast cell degranulation (27) and therefore explains the possible exacerbation experienced by CU patients (19, 28, 29). Furthermore, the direct viral cytopathic effect on keratinocytes through the ACE2 receptor of the basal layer of the epidermis has been postulated as an added hypothesis in the pathophysiology (27). It is important to emphasize the role of psychological stress faced by most people during the current sanitary crisis as a potential cause of these exacerbations since it has been previously demonstrated that there is a close association between CSU and anxiety (24, 26, 30). Although COVID-19 infection may result in flares of CSU in some patients, current evidence does not demonstrate that CSU is an independent predictor of the severity of COVID-19 infection or influences the course of the disease (24).

### Treatment considerations

Similar to patient with AD, patients with CSU reported great satisfaction with teledermatology (24, 25). The mainstay treatment for CSU consists of second-generation non-sedating H1 antihistamines, cyclosporine and omalizumab. There is no consensus between clinicians regarding the discontinuation of biologics or immune suppressants during the pandemic. Recently, the European Academy of Allergy and Clinical Immunology (EAACI) has published an expert-led consensus on practical recommendations for the management of patients with urticaria (24). Treatment with antihistamines should be maintained throughout the pandemic regardless of the infection status of the patient. As for cyclosporine, an immunosuppressant, there is no clear data whether it should be continued or be avoided (31). If the immunosuppressant cannot be replaced, it has been advised to decrease it to the lowest possible dose to avoid or minimize complications (31). Concerning omalizumab, expert opinion advises maintaining the treatment in patients with mild-to-moderate COVID-19 infection based on findings extrapolated from a meta-analysis of randomized controlled trials in CSU where similar rates of upper respiratory tract infections in omalizumab and placebo-treated individuals were detected (31, 32). Such recommendation has been substantiated by recent reports demonstrating the safety of omalizumab in COVID-19 patients with urticaria (28, 31, 33). In patients with severe COVID-19 infection, it has been agreed to prolong intervals between the omalizumab injections or pause therapy (24). Omalizumab is usually administered in office for the first third doses and home administration can be considered for the fourth. Early transition to home/self-administered omalizumab injections during the COVID-19 pandemic has been recently recommended to eliminate hospital visits and mitigate the risk of exposure to COVID-19 infection (34, 35). This can be achieved *via* training at the second dose which includes an action plan in the event of anaphylaxis (35, 36). Additionally, telemedicine may be provided to ensure proper patient education.

### Vaccination considerations

The literature is scarce regarding SARS-CoV-2 vaccination in patients with CSU. On one hand, a small case series reported flares of CSU induced by the mRNA vaccine (37). Most of these patients experienced a resolution of their symptoms within a week. Hence, it may be beneficial for this subset of patient to be evaluated by a physician prior to receiving the vaccine (37). On the other hand, the Canadian Society of Allergy and Clinical Immunology state that this condition is unlikely to interfere with the immune response triggered by the vaccine. Therefore, all patients including those maintained on omalizumab should proceed with their SARS-CoV-2 vaccine/s. Unfortunately, there is no consensus on what dosing schedule of omalizumab with respect to vaccine scheduling.

## Conclusion

The COVID-19 pandemic has largely affected clinicians' and patients' daily practices forcing both groups to quickly adapt to a new model of *modus operandi*. Current evidence indicates that patients with underlying skin allergies may be treated nearly like the general population. Special considerations should however be applied. These include modifying immune-modulating therapies and vaccination considerations. This sanitary emergency has also permitted the implementations of teledermatology on a large scale. Such a consultative platform is now widely accepted by patients with AD or CSU and provides continuity of care to these patients. The general recommendations published thus far and reviewed in this paper are largely derived from expert opinions and lack robust evidence for final recommendations. A collective worldwide effort is mandatory and is underway to obtain data from web-registries and design the appropriate management strategies needed in patients with skin allergies during this unabating pandemic.
